# Occurrence of Phlebotomine sand flies (Diptera: Psychodidae) in the northeastern plain of Italy

**DOI:** 10.1186/s13071-021-04652-2

**Published:** 2021-03-18

**Authors:** Alice Michelutti, Federica Toniolo, Michela Bertola, Marika Grillini, Giulia Simonato, Silvia Ravagnan, Fabrizio Montarsi

**Affiliations:** 1 Laboratory of Parasitology, Micology and Medical Entomology, Istituto Zooprofilattico Sperimentale Delle Venezie, Legnaro (PD), Italy; 2 Department of Animal Medicine, Production and Health, University of Padova, Legnaro (PD), Italy

**Keywords:** *Phlebotomus perniciosus*, *Phlebotomus mascittii*, *Phlebotomus perfiliewi*, *Sergentomyia minuta*, Lowland, Italy

## Abstract

**Background:**

Recent climate and environmental changes have resulted in the geographical expansion of Mediterranean *Leishmania infantum* vectors towards northern latitudes and higher altitudes in different European countries, including Italy, where new foci of canine leishmaniasis have been observed in the northern part of the country. Northern Italy is also an endemic area for mosquito-borne diseases. During entomological surveillance for West Nile virus, mosquitoes and other hematophagous insects were collected, including Phlebotomine sand flies. In this study, we report the results of Phlebotomine sand fly identification during the entomological surveillance conducted from 2017 to 2019.

**Methods:**

The northeastern plain of Italy was divided by a grid with a length of 15 km, and a CO_2_-CDC trap was placed in each geographical unit. The traps were placed ~ 15 km apart. For each sampling site, geographical coordinates were recorded. The traps were operated every two weeks, from May to November. Sand flies collected by CO_2_-CDC traps were identified by morphological and molecular analysis.

**Results:**

From 2017 to 2019, a total of 303 sand flies belonging to the species *Phlebotomus perniciosus* (*n* = 273), *Sergentomyia minuta* (*n* = 5), *P. mascittii* (*n* = 2) and *P. perfiliewi* (*n* = 2) were collected, along with 21 unidentified specimens. The trend for *P. perniciosus* collected during the entomological surveillance showed two peaks, one in July and a smaller one in September. Sand flies were collected at different altitudes, from −2 m above sea level (a.s.l.) to 145 m a.s.l. No correlation was observed between altitude and sand fly abundance.

**Conclusions:**

Four Phlebotomine sand fly species are reported for the first time from the northeastern plain of Italy. Except for *S. minuta*, the sand fly species are competent vectors of *Leishmania* parasites and other arboviruses in the Mediterranean Basin. These findings demonstrate the ability of sand flies to colonize new environments previously considered unsuitable for these insects. Even though the density of the Phlebotomine sand fly population in the plain areas is consistently lower than that observed in hilly and low mountainous areas, the presence of these vectors could herald the onset of epidemic outbreaks of leishmaniasis and other arthropod-borne diseases in areas previously considered non-endemic. 
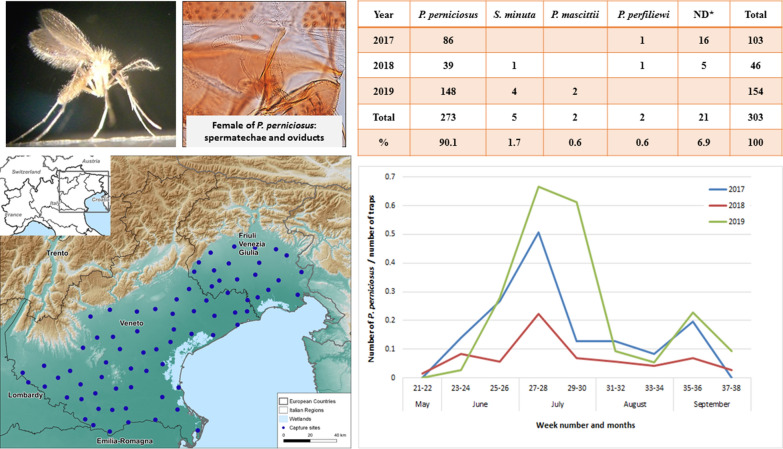

**Supplementary Information:**

The online version contains supplementary material available at 10.1186/s13071-021-04652-2.

## Background

Phlebotomine sand flies (Diptera: Psychodidae) are hematophagous insects and vectors of leishmaniasis and other bacterial and viral diseases [1–3]. In the Mediterranean region, they are the main vector of *Leishmania infantum*, the causative agent of canine leishmaniasis (CanL) and zoonotic human cutaneous (CL) and visceral leishmaniasis (VL) [2, 4, 5].

Different species of the genus *Phlebotomus* are implicated in the transmission of Mediterranean *Leishmania infantum*, including *Phlebotomus ariasi*, *P. balcanicus*, *P. kandelakii*, *P. langeroni*, *P. neglectus*, *P. perfiliewi*, *P. perniciosus*, *P. sergenti* and *P. tobbi* [2, 6, 7].

Phlebotomine sand fly abundance and distribution are closely related to climate and environmental factors [8–10]. Recent climate and environmental changes have resulted in the geographical expansion of Mediterranean *L. infantum* vectors towards northern latitudes and higher altitudes in different European countries [2, 6].

In Italy, CanL is endemic in the central and southern regions and the islands. Since the 1990s, new foci of CanL have been observed in the northern part of the country, previously considered non-endemic [11–17]. Entomological surveys conducted in these regions have confirmed the geographical expansion of Phlebotomine sand flies to northern latitudes: *P. perniciosus* was the most widespread species in both pre-Alpine and pre-Apennine territories, while *P. neglectus* was present only in pre-Alpine and *P. perfiliewi* in pre-Apennine sites [14]. These studies confirmed the most suitable habitat for these vectors is represented by hilly and low mountainous areas [11–13, 15, 18–20].

Among arthropod-borne diseases, mosquito-borne diseases (MBD) are endemic in northern Italy, in particular West Nile virus (WNV), a primarily avian virus, which has caused endemic outbreaks resulting in human and animal mortality [21]. After the first outbreak of West Nile disease [22], the Italian government developed an integrated surveillance plan that targeted birds, domestic poultry, horses, humans and mosquitoes [21], with the aim of early detection of viral circulation and reducing the risk of infection in humans [23, 24].

An entomological surveillance for WNV and other mosquito-borne viruses (Usutu, Dengue, Zika and mosquito-borne flaviviruses) was carried out by collecting host-seeking mosquitoes by traps. During the surveillance, other hematophagous insects were accidentally collected, including Phlebotomine sand flies. Following the first findings of sand fly specimens, we decided to keep them aside for identification. We did not perform any investigation to see whether the collected specimens were infected by *Leishmania* parasites, because VL, CL and CanL are notifiable diseases according to Italian Health Ministry regulations, and during the surveillance period there were no reported cases of *Leishmania* infection in the study area.

Herein, we report the results of Phlebotomine sand fly collection and identification during entomological surveillance for WNV conducted in the Friuli Venezia Giulia (FVG) and Veneto regions from 2017 to 2019.

## Materials and methods

### Study area

The study was conducted in the plain area (altitude below 300 m above sea level [a.s.l.]) of the FVG and Veneto regions. This area is characterized by a continental climate with hot summers and generally cold winters. Temperatures exceed 30 °C in summer and drop to values under −10 °C in winter, remaining under 0 °C even during the day. Precipitation levels range between 600 and 800 mm/year [19].

**Fig. 1:**
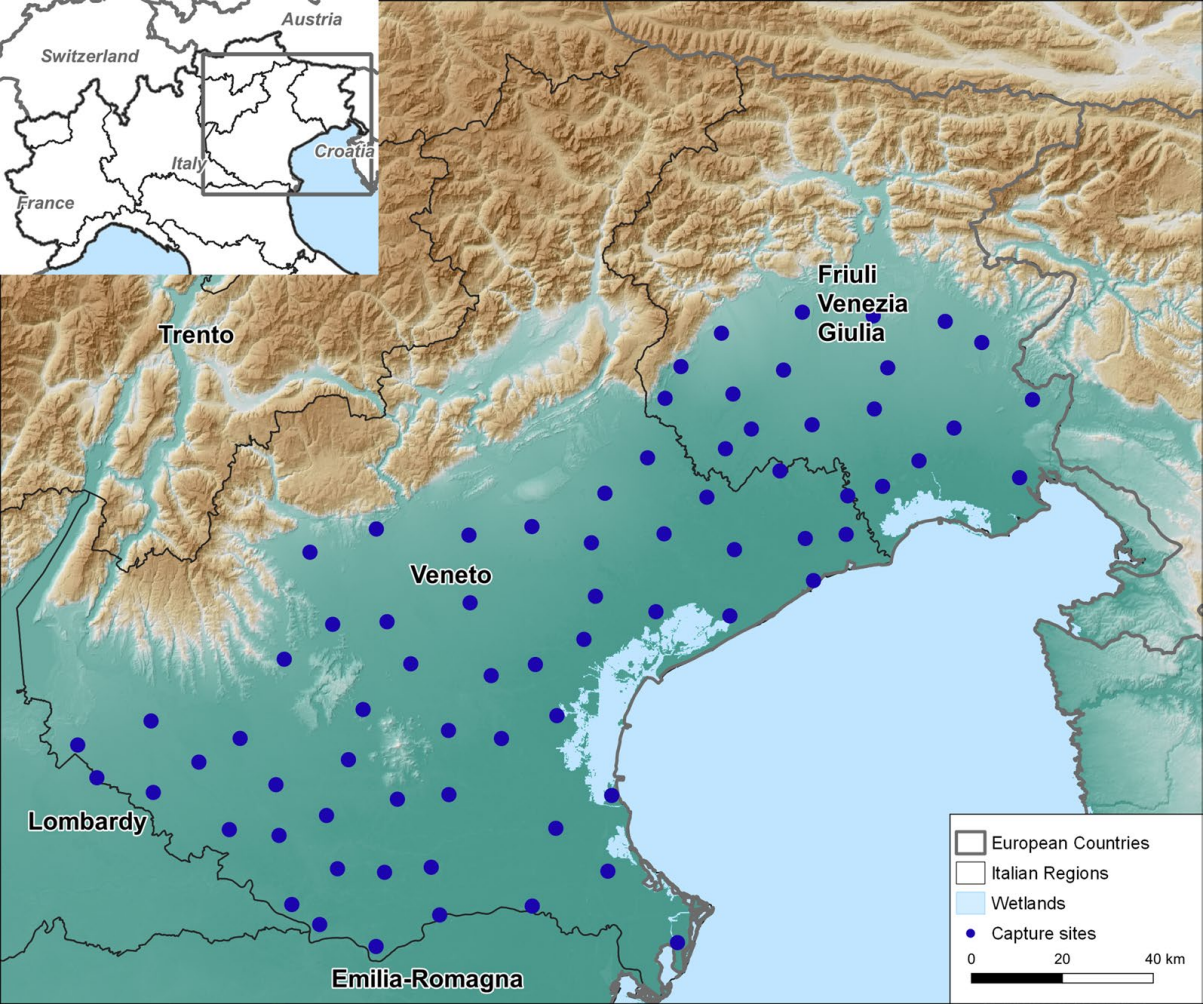
Geographical location of CO_2_-CDC traps in northeastern Italy in 2019

The area was divided into a grid with a length of 15 km. A trap for collecting host-seeking mosquitoes was placed in each geographical unit. The traps were placed ~ 15 km apart. The number of traps was adjusted each year according to WNV epidemiology, with 71, 72 and 75 traps in operation in 2017, 2018 and 2019, respectively. An example of the distribution of the traps is reported in Fig. 1. Waypoints were included in the map using QGIS software [25].

The geographical coordinates and a brief description of the environment (farm, rural or urbanized area) were recorded for each site.

### Sand fly collection and identification

Collections were carried out using a CO_2_-CDC trap (IMT^®^—Italian Mosquito Trap, Cantù, Italy). CDC traps were filled with dry ice pellets as a source of carbon dioxide in order to attract hematophagous insects and powered by a 12-V battery. Traps were operated from sunset to sunrise, every two weeks. The entomological surveillance started each year from the second week of May and continued until the first week of November. Insects were delivered to the laboratory in dry ice, to preserve the integrity of the viral RNA in mosquito vectors.

From 2017 to 2019, all collected sand flies were systematically preserved in 70% ethanol and morphologically identified at the end of the monitoring season. In addition to morphological identification, sand flies collected in 2019 were also identified by molecular assay.

For morphological identification, the heads and genitalia of each sand fly were dissected, clarified using chloral hydrate and acetic acid, and slide-mounted in Hoyer’s solution, as described by Dantas-Torres et al. [26]. Sand flies were examined with an optical microscope and identified using morphological keys [26] based on specific features of the pharynx and genitalia (spermathecae in females and external genitalia in males).

For molecular identification, the thorax, wings and legs were maintained on a petri dish at room temperature (20 °C) for at least 1 hour and rinsed twice with phosphate-buffered solution (PBS) to remove the alcohol. After rinsing, the specimens were transferred to a 2 ml Eppendorf^®^ tube with a sterile steel ball and stored at −20 °C, before proceeding with DNA extraction. DNA was extracted from each specimen using the MagMAX™ Pathogen RNA/DNA Kit (Thermo Fisher Scientific) and Hamilton Microlab STARlet automated extraction instrument. Amplification and sequencing were performed according to the procedure described by Jalali et al. [27].

### Statistical analysis

Since the number of Phlebotomine sand flies other than *P. perniciosus* was very low, this species was considered for statistical analysis. Linear regression was carried out with the independent variable (altitude) plotted against the abundance of *P. perniciosus* as the dependent variable, while the chi-square test was used to compare the percentages of environments positive for *P. perniciosus*.

## Results

The geographical coordinates (longitude, latitude and altitude) for each sampling site and a brief description of the environment (farms, rural or urbanized area) are reported in Additional file 1: Table S1.

Regarding species identification, a total of 303 sand flies belonging to the species *P. perniciosus* (*n* = 273), *S. minuta* (*n* = 5), *P. mascittii* (*n* = 2) and *P. perfiliewi* (*n* = 2) were collected. Morphological identification of 21 specimens (collected in 2017 and 2018) was not possible due to damage to anatomical structures needed for this purpose (Table 1).

**Table 1:** Species and number of Phlebotomine sand flies collected per year

Year	*P. perniciosus*	*S. minuta*	*P. mascittii*	*P. perfiliewi*	ND	Total
2017	86			1	16	103
2018	39	1		1	5	46
2019	148	4	2			154
Total	273	5	2	2	21	303
%	90.1	1.7	0.6	0.6	6.9	100

*ND* not determined

Molecular identification, performed on all 154 specimens collected in 2019, confirmed the results of morphological identification (Table 1) and it was very helpful for the identification of damaged samples.

**Fig. 2:**
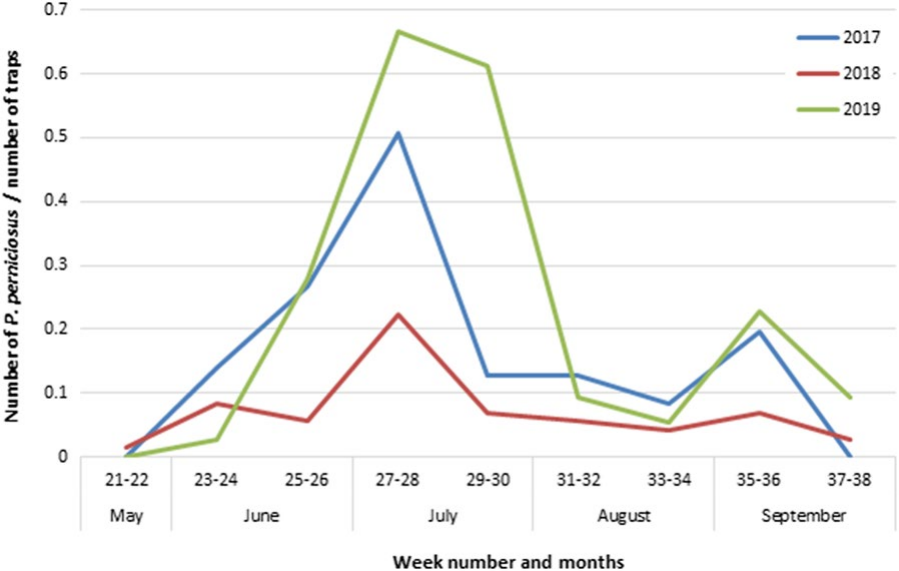
Trend of *P. perniciosus* in 2017, 2018 and 2019

Sand flies were collected from late May to September, with a peak in July (weeks 27–28 in 2017 and 2018, weeks 27–30 in 2019) and a smaller peak in September (weeks 35–36 in 2017 and 2019). The trend for *P. perniciosus* collected during entomological surveillance is shown in Fig. 2.

Sand flies were collected at different altitudes, from −2 m a.s.l. (Porto Viro, Rovigo) to 145 m a.s.l. (Thiene, Vicenza). No correlation was observed (correlation coefficient = 0.20) between altitude and *P. perniciosus* abundance, as shown in Additional file 2: Figure S1.

Sand flies were collected from farms, rural and urbanized areas. The number of sites positive for Phlebotomine sand flies for each environmental type is reported in Additional file 3: Table S2.

## Discussion

In this study, we report for the first time the presence of Phlebotomine sand flies in the northeastern plain of Italy. Although other studies have reported sand flies in these regions, they were collected from hilly and low mountainous areas [11–13, 15, 18–20]. An increase in the distribution area of sand flies was observed only in the neighboring region, Emilia-Romagna, where *P. perfiliewi* and *P. perniciosus* were collected from the whole regional territory [28, 29].

In this study, *P. perniciosus* was the dominant species, while *P. neglectus* was not isolated during the entomological surveillance, although it is considered quite a common species in northern Italy [11–14, 18, 19]. *Phlebotomus mascittii* was observed for the first time in FVG (Premariacco, 112 m a.s.l.), and *P. perfiliewi* was detected for the first time in the FVG (Caneva, 33 m a.s.l.) and Veneto regions (Badia Polesine, 8 m a.s.l.). *Sergentomyia minuta* was also reported for the first time in the FVG region, in the village of Budoia (135 m a.s.l.); this species was previously found in the Veneto region in the locality of Calaone (178 m a.s.l.) [18, 19], but in this study it was observed in sites at lower altitudes, such as Porto Viro (2 m a.s.l.) and Albignasego (8 m a.s.l.).

Since *S. minuta* is a herpetophilic sand fly, its role in the transmission of *L. infantum* is not important [30], unlike *P. perfiliewi*, *P. mascittii* and *P. perniciosus*.

It is noteworthy that *P. perfiliewi* had never been reported in these regions, although it is the most abundant species in the neighboring region (Emilia Romagna) [31] and in central [32] and southern Italy [33].

*Phlebotomus perfiliewi* is a proven vector of *L. infantum* and its presence was documented in several outbreaks of CanL in the Emilia Romagna region [28, 29]. The presence of *P. perfiliewi* was also observed in outbreaks of human VL, caused by *L. infantum* strains other than those isolated from dogs [31]. This finding suggests the involvement of *P. perfiliewi* in a sylvatic cycle of leishmaniasis in which wildlife plays the role of reservoir [34] rather than dogs [31].

In the Emilia Romagna region, *P. perfiliewi* is a vector of the Toscana virus (TOSV) and other phleboviruses [35, 36] and it was also found to be positive for a trypanosome related to *Trypanosoma theileri* (Kinetoplastea: Trypanosomatidae) [37]. However, in the FVG and Veneto regions, *P. perfiliewi* abundance is so limited that it is unlikely to play a role as vector of *Leishmania* parasites or arboviruses.

*Phlebotomus mascittii* is not a proven vector of leishmaniasis [38], although recent detection of *L. infantum* DNA in specimens of *P. mascittii* from Austria [39] and Italy [40] may support possible competence in the transmission [41]. Thus, its role as *Leishmania* vector should be considered, at least in areas where it is abundant and *Leishmania*-infected dogs are imported from endemic countries [42].

*Phlebotomus perniciosus* is an efficient vector of *L. infantum* in Italy [43] and in southwestern European countries [6]. In experimental conditions, it is a competent vector of *L. tropica*, causing CL [41, 44]. It is also a vector of arboviruses such as TOSV [45].

In the study area, the trend in *P. perniciosus* during the entomological surveillance was bimodal, with a peak at the beginning and the end of the warm season. A bimodal trend in *P. perniciosus* was observed in Mediterranean sites located at low latitudes; at intermediate or higher latitudes, single large peaks or two partially confluent peaks have been recorded [6]. However, there are examples of variable *P. perniciosus* distribution patterns even at higher latitudes and a sharp bimodal trend, attributable to climate warming, was recently reported in southern France. Climate change might enhance the risk of pathogen transmission because of the higher sand fly density and the longer activity season [46].

It is common knowledge that the distribution of sand flies is heavily influenced by altitude [47].

Considering the difference in sand fly abundance, the number of specimens collected in 2019 was higher than in previous years because of the addition of new sites, with a higher number of sand flies collected.

In northern Italy, *P. perniciosus* was detected at high densities in hilly and low mountains sites [14]. This finding was confirmed by the ecological niche model described by Signorini et al. [19], where the highest relative probability of *P. perniciosus* occurrence was predicted in hilly areas between 100 and 300 m a.s.l. In our study, *P. perniciosus* was also present in the lowlands at 7 m a.s.l. (Brugine, Veneto region), indicating that altitude alone is an inadequate predictor variable of sand fly abundance [48]. Our findings also suggest an adaptation of sand flies to new environments, characterized by low-altitude sites with a continental climate and peri-urban environment.

The ability of Phlebotomine sand flies to adapt to environmental changes was observed in southern France, where Prudhomme et al. [49] observed that the sand fly population did not differ significantly from what was reported by previous studies conducted in the same area, even though the habitat has been transformed by urbanization, with reduced host abundance and increased temperature over the years.

The adaptation of Phlebotomine sand flies to human dwellings was also observed in southern Italy, enhancing the potential risk of exposure to *L. infantum* among local inhabitants [33].

Certainly, some critical points are identified in the collection method: although CO_2_-CDC traps are considered more effective than others (i.e. sticky traps or CDC-light traps) in collecting sand flies [18], sampling sites were not located in areas suitable for these insects. Therefore, entomological surveillance focusing on sand fly detection is needed to have a more realistic picture of its distribution in the plain area.

The detection of sand flies in new areas traditionally considered as non-endemic represents important data for public health. In light of the growing movement of dogs from or to endemic areas (adoptions or tourism) [42], in addition to a proper awareness among veterinarians and public health authorities, dog owners should be aware of the health risk and should be adequately educated in the correct use of preventive measures against sand fly bites. Although outbreaks of *L. infantum* are more frequently described in non-endemic areas, dog owner education programs on the correct use of preventive measures against sand fly bites are found to achieve successful results in controlling the spread of the infection [12, 20].

## Conclusions

Phlebotomine sand flies are reported for the first time from the northeastern plain of Italy. Except for *S. minuta*, the sand fly species are competent vectors of *L. infantum* and other arboviruses in the Mediterranean Basin.

Although the density of the Phlebotomine sand fly population in the plain areas is consistently lower than that observed in hilly and low mountainous areas, the presence of the vector in these environments could herald the onset of epidemic outbreaks of leishmaniasis and other diseases in areas previously considered non-endemic.

## Supplementary Information

**Additional file 1: Table S1.** List of sampling sites, with geographical coordinates and environmental description and monitoring years.

**Additional file 2: Figure S1. **Scatter plot of altitude (m) and mean number of *P. perniciosus*.

**Additional file 3: Table S2. **Number of sites positive for Phlebotomine sand flies in each environmental type.

## Data Availability

The data supporting the conclusions of this article are included within the article and its additional file.

## Abbreviations

CanL: Canine leishmaniasis
CL: Cutaneous leishmaniasis
VL: Visceral leishmaniasis
MBD: Mosquito-borne diseases
WNV: West Nile virus
FVG: Friuli Venezia Giulia
a.s.l.: Above sea level
