# Supplementary figures and images for: Occurrence of Phlebotomine sand flies (Diptera: Psychodidae) in the northeastern plain of Italy
Source: Parasit Vectors. 2021 Mar 18;14:164. doi: 10.1186/s13071-021-04652-2 (PMC7992963; doi:10.1186/s13071-021-04652-2)

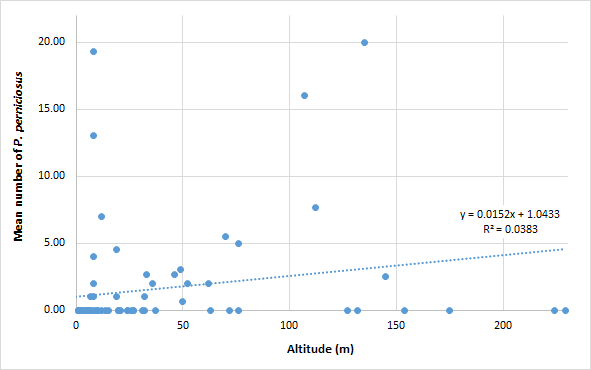

Supplement: Supplementary file 2 — Additional file 2: Figure S1. Scatter plot of altitude (m) and mean number of P. perniciosus. [file 13071_2021_4652_MOESM2_ESM.png]
